# Supplementary material for: MetaRibo-Seq measures translation in microbiomes
Source: Nat Commun. 2020 Jun 29;11:3268. doi: 10.1038/s41467-020-17081-z (PMC7324362; doi:10.1038/s41467-020-17081-z)
Supplement: Supplementary file 10 — Supplementary Data 7 [file 41467_2020_17081_MOESM10_ESM.zip › File2/Confidence_High_Taxonomy/86381_out.krona.html]

Javascript must be enabled to view this page.

members
magnitude
magnitudeUnassigned
count
unassigned
taxon
rank

86381\_out

3

3
2
superkingdom

phylum
1
1239

class
1
186801

order
186802
1

family
1
541000

1
216851
genus


SRS014948\_contig\_number\_2872
species
853
1

2
201174
phylum

2
1760
class

order
2
85004

31953
2
family

1678
2
genus


SRS077849\_contig\_number\_contig-100\_14711.14711
species
1680
1

41200
1
species

SRS019068\_contig\_number\_contig-100\_9598.442846
